# Myasthenic Crisis In Pregnancy

**DOI:** 10.5811/cpcem.2017.5.33404

**Published:** 2017-10-03

**Authors:** David M. French, E. Page Bridges, Matthew C. Hoskins, Charles M. Andrews, Cecil H. Nelson

**Affiliations:** * Medical University of South Carolina, Emergency Medicine, Charleston, South Carolina; † Greenville Health System, Emergency Medicine, Greenville, South Carolina; ‡ Medical University of South Carolina, Neurocritical Care, Charleston, South Carolina; § Medical University of South Carolina, Department of Obstetrics and Gynecology, Charleston, South Carolina

## Abstract

This case reviews the management of a 27-year-old pregnant female in myasthenic crisis. She presented to the emergency department in respiratory distress refractory to standard therapy, necessitating airway and ventilatory support and treatment with plasmapheresis. Myasthenic crisis in the setting of pregnancy is rare and presents unique management challenges for emergency physicians.

## INTRODUCTION

Myasthenia gravis (MG) is an autoimmune neuromuscular disorder characterized by skeletal muscle weakness that worsens with repetitive use and improves with rest.^1^ It is a T-cell dependent, B-cell mediated disease, most commonly caused by production of auto-antibodies against post-synaptic skeletal muscle nicotinic acetylcholine receptors (AChR) at the neuromuscular junction (NMJ). They may have antibodies to muscle-specific tyrosine kinase receptors (MuSK).^2^,^3^ Thymomas are frequently associated with MG and can have atypical autoantibodies.^4^ The prevalence is 5–15/100,000 people and is twice as common in women.^5^ MG can be challenging in pregnancy, given its typical occurrence during the third decade of life.^6^

## CASE REPORT

A 27-year-old 58-kilogram Caucasian female presented to the emergency department (ED) with acute onset cough, increased secretions, and dyspnea. She had a history of asthma, pulmonary embolism and double seronegative myasthenia gravis (MG) status post thymectomy and was gravida one para zero at 31-weeks gestation. Her MG had been refractory to standard medical therapies during pregnancy, necessitating scheduled plasmapheresis every three weeks. Additional management included systemic corticosteroids and Lovenox injections, though previous hypercoagulable testing had been negative. Her pregnancy had been uncomplicated except for mild MG symptoms of ptosis and difficulty with mastication.

Further review of systems revealed nasal congestion for three days with sore throat but no fever. She denied chest pain or edema but did complain of resting dyspnea and generalized weakness, similar to past myasthenic flares. That evening, she had increasing difficulty clearing her secretions despite her cough, and albuterol treatment by emergency medical services did not improve her symptoms.

On exam, she was in respiratory distress with accessory muscle usage. Vital signs were heart rate 120 beats/minute, blood pressure 120/84 millimeters of mercury (mm Hg), respiratory rate 26 breaths/minute, oxygen saturation 100% on 35% fraction of inspired oxygen by facemask, and temperature 36.7 degrees Celsius. She had stridor on exam, but otherwise heart and lungs were normal. Her abdomen was gravid but nontender. The remainder of her physical exam was unremarkable. Chest radiograph was normal. Her initial maximum inspiratory pressure (MIP) was -15 centimeters of water (cm H_2_O) and forced vital capacity (FVC) was 2.0 liters, with an arterial blood gas (ABG) showing pH 7.54, partial pressure of carbon dioxide (PaCO_2_) 21.5 mm Hg, bicarbonate 18 milliequivalents/liter (mEq/L), partial pressure of oxygen (PaO_2_) 177 mm Hg.

Given her poor respiratory status and worsening fatigue, the patient was trialed on bilevel positive airway pressure (BiPap) but had minimal improvement, so she was intubated without complications. A computed tomography pulmonary angiogram demonstrated a small, clinically insignificant subsegmental pulmonary embolism. Testing for rhino/enterovirus was positive, bilateral lower extremity Dopplers were normal, and the patient was admitted to the intensive care unit for myasthenic crisis and respiratory failure.

She was started on pyridostigmine and dexamethasone for MG and fetal lung maturity. Intravenous immunoglobulin (IVIg) was previously ineffective, so she underwent plasmapheresis every other day for five treatments. She was successfully extubated and transferred to the antepartum service, but her course was further complicated by hemolysis, elevated liver enzymes and low platelet count (HELLP syndrome) with rising blood pressure, necessitating cesarean section. Magnesium was avoided for fear of worsening her MG. A vigorous female infant was delivered without evidence of neonatal MG or arthrogryposis and was discharged after one month of management for prematurity. The mother also recovered completely.

## DISCUSSION

In the initial evaluation of a patient with weakness and suspected MG, history and physical exam are critical. Patients typically present with painless, fluctuating weakness of the skeletal muscles.^7^ Classically, the extra-ocular muscles are involved, leading to ptosis and diplopia, but bulbar symptoms may be seen, such as difficulty with mastication, swallowing, and speaking. Other symptoms can include proximal trunk and limb (upper more than lower) muscle weakness.^8^ While most patients present with ocular symptoms, many progress to generalized myasthenia within two years.^9^ The diagnosis is based on clinical symptoms but can be confirmed with electromyography (EMG) or with improvement of symptoms after administration of anti-cholinesterase medications (edrophonium).^10^ Such testing is not recommended in unstable patients, as there is a significant risk of false positive or negative results and potential for worsening symptoms.^5^ In patients without known diagnosis of MG, the differential includes neuromuscular diseases such as Lambert-Eaton, Guillain-Barre, botulism, overdose, tick paralysis, or intracranial pathology, as well as anticholinergic crisis for patients taking cholinesterase-inhibiting medications, such as pyridostigmine.^1^,^3^,^5^,^11^

In 15% of patients, life-threatening respiratory muscle weakness of the diaphragmatic, intercostal, and abdominal muscles, combined with an inability to maintain secretions can result in respiratory failure and is termed myasthenic crisis (MC).^1^,^3^,^5^,^9^ Most patients presenting with MC have a predisposing factor, usually a respiratory infection, though emotional stress, pregnancy, thyroid disease, electrolyte abnormalities, surgery, trauma, and medication changes can trigger a crisis.^5^,^12^,^13^ Without prompt intervention, patients with MC may have rapid deterioration of respiratory status, and early intubation is critical.^1^

Respiratory testing can help guide the decision to intubate, and the “20/30/40” rule is a commonly cited tool.^5^ This refers to FVC < 20 milliliters/kilogram, maximum inspiratory pressure (MIP) or negative inspiratory force (NIF) < −30 cm H_2_O, and maximum expiratory pressure (MEP) < 40 cm H_2_0.^12^ Other factors, such as neck flexor muscle weakness with inability to raise the head, difficulty handling secretions, and paradoxical muscle weakness, should also be considered.^3^,^14^ The ability to hold the head off the bed for five seconds and swallow five milliliters of liquid or count to 20 in one respiratory cycle are subjective but make respiratory failure unlikely.^14^,^15^ As patients with MC can experience rapid respiratory failure, close clinical monitoring of patients with frequent reassessment of respiratory status is essential. ABGs are less important than clinical parameters, as changes to PaO_2_ and PaCO_2_ are later findings in MC.^15^

**CPC-EM Capsule**

What do we already know about this clinical entity?

*Myasthenic crisis is rarely treated in emergency departments, though there are some emergency medicine articles on presentations of myasthenia gravis and management of myasthenic crisis*.

What makes this presentation of disease reportable?

Myasthenic crisis is even more rarely seen during pregnancy and presents unique management challenges due to associated physiologic changes and limited treatment options.

What is the major learning point?

Evaluation of airway and ventilatory status are essential in the management of myasthenic crisis, and early intubation may be critical.

How might this improve emergency medicine practice?

This article reviews assessment tools and management techniques to successfully treat myasthenic crisis, with special consideration given to pregnancy.

In contrast to Guillain-Barre, noninvasive positive pressure ventilation may be attempted before intubation. In one retrospective cohort study, 14 of 24 patients in MC were successfully treated with BiPAP. The only predictor of BiPAP failure in this study was a PaCO_2_ level > 45 mm Hg when BiPAP was initiated.^16^

If intubation is required, medications to facilitate airway management should be chosen carefully. The NMJ during myasthenic crisis has a decreased number of functional AChRs, and paralytic agents have a different and sometimes unpredictable response. Depolarizing agents, such as succinylcholine, have resistance at the NMJ, resulting in a median effective dose (ED_50_) and dose required for effect in 95% of the population (ED_95_) that are 2.0 and 2.6 times normal, respectively.^17^ In other words, much higher doses of succinylcholine may be needed for rapid sequence intubation, with estimates of 2.6 times the usual dose.^18^ Simultaneously, many MG patients are medicated with anticholinesterase medications, such as pyridostigmine, which decrease plasma cholinesterase activity, leading to decreased hydrolysis of succinylcholine and prolonged neuromuscular blockade.^14^ This decrease in NMJ receptors also makes MG patients very sensitive to nondepolarizing drugs. For example, the dose of vecuronium needed for paralysis is approximately 0.4 to 0.55 times the usual dose (approximately 0.05 milligrams/kilogram).^18^

Ultimately, the mainstays of treatment of MC are plasma exchange (PE) or IVIg,^19^ which have similar efficacy.^20^,^21^ PE requires one exchange every other day for 10 days, while IVIg is usually administered for five days. Concurrent use is avoided, as PE may remove IVIg; however, these therapies can be given sequentially, if the patient demonstrates inadequate response to therapy. High dose prednisone, 60–100 milligrams daily, can also be initiated, with effects usually seen after two weeks.^12^

As with most auto-immune diseases in pregnancy, MG symptoms will improve in 30% of patients, remain stable in 30%, and worsen for 40%.^22^,^23^ Physiologic changes of pregnancy also make MG management difficult. While maternal respiratory rate remains constant, there is a 40% increase in tidal volume with a concomitant decrease in expiratory reserve volume and residual volume, resulting in baseline maternal hypocapnia and hyperventilation. This leads to baseline respiratory alkalosis, leaving less reserve in myasthenic crisis.

There is also an increased risk of preterm birth in the setting of congenital myasthenia; however, this is linked to the resultant polyhydramnios caused by the loss of fetal swallowing.^22^ Myasthenia has not been found to increase the risk of miscarriage, growth restriction or pre-eclampsia.^24^ However, in pre-eclampsia or eclampsia, magnesium sulfate is contraindicated, as it affects the NMJ, hindering acetylcholine release and worsening MG. If magnesium must be used, providers should monitor for respiratory depression and be prepared to provide immediate ventilatory support, if needed. Phenytoin and diazepam are alternatives to magnesium in these cases but are not as effective.^25^

General management of MG in pregnancy involves standard therapy, including acetylcholinesterase inhibitors, corticosteroids, and various immunosuppressants.^26^ Only a few medications should be avoided due to teratogenic potential during pregnancy, including methotrexate and mycophenolate mofetil;^27^ however, these medications are typically used in disease maintenance and not for acute exacerbation. Corticosteroids have been associated with a substantial improvement or remission in almost 80% of patients.^28^ In the setting of myasthenic crisis, IVIg and plasmapheresis may be used, though IVIg therapy is preferable, as plasmapheresis may cause maternal hypotension and decreased placental perfusion.

The principal neonatal concerns in the setting of myasthenia include transient neonatal myasthenia gravis (TNMG) and arthrogryposis multiplex congenita (AMC). TNMG is caused by maternal antibodies crossing the placenta during the third trimester, which may affect 9–30% of pregnancies.^22^,^29^,^30^ The risk does not correlate with maternal disease severity.^31^ The symptoms of TNMG, include weak tone/cry, hypotonia, poor suck, ptosis, and respiratory problems, and typically develop over 12 hours to several days following delivery. Treatment is supportive, with nasogastric tube feeding, respiratory support, and oral or intravenous acetyl-cholinesterase inhibitors. Only severe cases require IVIg or PE.

In rare cases of myasthenia gravis, maternal antibody production is against the fetal gamma subunit of the AChR, leading to AMC. This condition has a constellation of neonatal findings, including non-progressive multiple joint contractures, small palate and jaw, and lung hypoplasia that often leads to neonatal or perinatal death.^32^ The most significant antenatal findings are polyhydramnios and evidence of limb contractures on ultrasound. AMC may occur in women who have minimal symptoms, so serial ultrasounds are recommended throughout pregnancy.

## CONCLUSION

While some emergency medicine literature on myasthenia gravis exists, myasthenic crisis is rarely seen in pregnancy and presents unique management challenges for emergency physicians. Close clinical monitoring of airway and ventilation are critical. However, practitioners should also consider longer term interventions that may requi**re** transfer to specialty care**.**

## References

[1] Smulowitz PB, Zeller J, Sanchez LD (2005). Myasthenia gravis: Lessons for the emergency physician. Eur J Emerg Med.

[2] Mays J, Butts CL (2011). Intercommunication between the neuroendocrine and immune systems: Focus on myasthenia gravis. Neuroimmunomodulation.

[3] Chaudhuri A, Behan PO (2009). Myasthenic crisis. QJM.

[4] Romi F (2011). Thymoma in myasthenia gravis: from diagnosis to treatment. Autoimmune Dis.

[5] Godoy DA, Vaz de Mello LJ, Masotti L (2013). The myasthenic patient in crisis: An update of the management in neurointensive care unit. Arq Neuropsiquiatr.

[6] Phillips LH (1994). The epidemiology of myasthenia gravis. Neurol Clin.

[7] Golden SK, Reiff CJ, Painter CJ (2015). Myasthenia gravis presenting as persistent unilateral ptosis with facial droop. J Emerg Med.

[8] Graves M, Katz JS (2004). Myasthenia gravis. Curr Treat Options Neurol.

[9] Statland JM, Ciafaloni E (2013). Myasthenia gravis: Five new things. Neurol Clin Pract.

[10] Howard JF (2013). Electrodiagnosis of disorders of neuromuscular transmission. Phys Med Rehabil Clin N Am.

[11] Ganti L, Rastogi V (2016). Acute generalized weakness. Emerg Med Clin N Am.

[12] Wendell LC, Levine JM (2011). Myasthenic crisis. Neurohospitalist.

[13] Van Berkel MA, Twilla JD, England BS (2016). Emergency department management of a myasthenia gravis patient with community-acquired pneumonia: does initial antibiotic choice lead to cure or crisis?. J Emerg Med.

[14] Baraka A (1992). Anaesthesia and myasthenia gravis. Can J Anaesth.

[15] Ferguson IT, Murphy RP, Lascelles RG (1982). Ventilatory failure in myasthenia gravis. J Neurol Neurosurg Psychiatry.

[16] Seneviratne J, Mandrekar J, Wijdicks EF (2008). Noninvasive ventilation in myasthenic crisis. Arch Neurol.

[17] Eisenkraft JB, Book WJ, Mann SM (1988). Resistance to succinylcholine in myasthenia gravis: a dose-response study. Anesthesiology.

[18] Abel M, Eisenkraft JB (2002). Anesthetic implications of myasthenia gravis. Mt Sinai J Med.

[19] Mantegazza R, Bonanno S, Camera G, Antozzi C (2011). Current and emerging therapies for the treatment of myasthenia gravis. Neuropsychiatr Dis Treat.

[20] Gajdos P, Chevret S, Clair B (1997). Clinical trial of plasma exchange and high-dose intravenous immunoglobulin in myasthenia gravis. Myasthenia Gravis Clinical Study Group. Ann Neurol.

[21] Barth D, Nabavi Nouri M, Ng E (2011). Comparison of IVIg and PLEX in patients with myasthenia gravis. Neurology.

[22] Batocchi AP, Majolini L, Evoli A (1999). Course and treatment of myasthenia gravis during pregnancy. Neurology.

[23] Ferrero S, Pretta S, Nicoletti A (2005). Myasthenia gravis: management issues during pregnancy. Eur J Obstet Gyncecol Reprod Biol.

[24] Varner M (2013). Myasthenia gravis and pregnancy. Clin Obstet Gynecol Reprod Biol.

[25] Walss Rodriguez RJ, Reyes Levario A (1992). Ginecol Obstet Mex.

[26] Gurjar M, Jagia M (2005). Successful management of pregnancy-aggravated myasthenic crisis after complete remission of the disease. Aust N Z J Obstet Gynaecol.

[27] Sifontis NM, Coscia LA, Constantinescu S (2006). Pregnancy outcomes in solid organ transplant recipients with exposure to mycophenolate mofetil or sirolimus. Transplantation.

[28] Evoli A, Batocchi AP, Palmisani MT, Lo Monaco M, Tonali P (1992). Long-term results of corticosteroid therapy in patients with myasthenia gravis. Eur Neurol.

[29] Djelmis J, Sostarko M, Mayer D (2002). Myasthenia gravis in pregnancy: Report on 69 cases. Eur J Obstet Gynecol Reprod Biol.

[30] Podciechowski L, Brocka-Nitecka U, Dabrowska K (2005). Pregnancy complicated by myasthenia gravis – twelve years experience. Neuro Endocrinol Lett.

[31] Hantaï D, Richard P, Koenig J (2004). Congenital myasthenic syndromes. Curr Opin Neurol.

[32] Vincent A, Newland C, Brueton L (1995). Arthrogryposis multiplex congenita with maternal autoantibodies specific for a fetal antigen. Lancet.

